# Anatomical Changes After Endoscopic Sinus Surgery in Patients with Chronic Rhinosinusitis

**DOI:** 10.3390/jcm14072380

**Published:** 2025-03-30

**Authors:** Ameen Biadsee, Rabie Shehadeh, Matan Katz, Tomer Boldes, Taciano Rocha, Brian W. Rotenberg, Leigh J. Sowerby

**Affiliations:** 1 Department of Otolaryngology—Head and Neck Surgery, Western University, London, ON N6A 3K7, Canada; rshehadeh1@gmail.com (R.S.); rocha.taciano@gmail.com (T.R.); brian.rotenberg@sjhc.london.on.ca (B.W.R.); leigh.sowerby@sjhc.london.on.ca (L.J.S.); 2 Department of Otorhinolaryngology—Head and Neck Surgery, Meir Medical Center, Kfar-Saba 4428164, Israel; matan.katz@clalit.org.il (M.K.); tomerboldes@gmail.com (T.B.); 3 School of Medicine, Faculty of Medical and Health Sciences, Tel Aviv University, Tel Aviv 6997801, Israel

**Keywords:** functional endoscopic surgeries, ethmoidectomy, enophthalmos, inferior turbinate, outfracture of the inferior turbinate, nasal airway

## Abstract

**Background:** Changes in the bony structures of the nose and sinuses such as the medialization of the lamina papyracea and enophthalmos have been reported after sinus surgery. Evidence for the persistence of inferior turbinate (IT) position after IT outfracture is lacking. **Objectives:** To evaluate for anatomical changes of the IT, lamina and the globes, after sinus surgery and the durability of inferior turbinate outfracture. **Methods:** A total of forty-four patients who underwent revision endoscopic sinus surgery that included complete ethmoidectomy and IT outfracture were matched. Pre- and post-operative computed tomography scans (CT) were used for evaluating and measuring the anatomical changes in different planes. The posterior globe position in the axial plane, the distance between the lamina papyracea (IOD_Axial_, IOD_Coronal_) in coronal and axial planes and the distance from the IT to the septum (IT_M_) and the lateral nasal wall (IT_L_) were measured. **Results:** There were 16 women and 28 men. Mean follow-up time (time from procedure to post-operative CT scan) was 38.9 ± 20.1 months. Statistically significant lateralization of the IT was observed with IT_L_ (95%CI 1.1 mm to 1.5 mm *p* < 0.0001) and IT_M_ (95%CI −1.5 mm to −1.1 mm; *p* < 0.0001). No statistically significant differences were seen in IOD_Axial_ and IOD_Coronal_ in pre-op and post-op CT scans. (*p* = 0.23 and *p* = 0.7, respectively) and no significant displacement of the globe in antero-posterior direction was seen (*p* = 0.915). **Conclusions:** IT outfracture appears to have a durable effect on IT position that lasts for several years. Ethmoidectomy did not cause the medialization of the laminae nor altered the position of the globes.

## 1. Introduction

The inferior turbinate is an important anatomical structure within the nasal cavity, composed of a thin bony framework (the turbinate or conchal bone) covered by highly vascularized respiratory mucosa. Its anterior segment attaches to the maxilla and its posterior segment to the palatine bone, forming a baffle that regulates airflow. The submucosal cavernous venous plexus of the IT, under autonomic control, allows the turbinate to engorge or decongest, thereby modulating nasal resistance as part of the normal nasal cycle [1,2,3]. Disruption of its anatomy through surgery may alter nasal aerodynamics, underscoring the importance of understanding long-term structural outcomes [4]. Hypertrophy of the IT—whether of the bony component, the mucosa or both—is a common contributor to chronic nasal obstruction in conditions like allergic rhinitis. In such cases, surgical reduction of turbinate size or the lateralization of its position is often necessary to restore airflow [5]. Outfracture of the inferior turbinate (IT) can be performed during nasal surgeries. Lateralization of the IT allows better access to the middle meatus, and improves nasal breathing by enlarging the nasal airway. The effect of outfracture is well established in several radiological studies [6,7,8,9]. However, the duration of this effect has only been reported for 6–9 months after surgery.

Moreover, IT outfracture reduces airflow resistance and can improve inspiratory airflow distribution. Computational fluid dynamics (CFD) simulations have shown that reducing the bulk of the inferior turbinate or moving it laterally leads to a broad reduction in intranasal airflow pressure and resistance, with increased airflow in the lower nasal cavity [10].

The lamina papyracea (LP), in contrast, is a paper-thin bony plate that forms the lateral wall of the ethmoid sinus and the medial wall of the orbit. It gains much of its structural support from the contiguous ethmoid bony partitions (ethmoid septa) and its peripheral bony sutures. During ESS, these internal ethmoid struts are removed, leaving the LP as an isolated plate between the now-enlarged ethmoid cavity and the orbital contents. Theoretically, the loss of ethmoidal support could predispose the LP to medialization (bowing inward toward the sinus) [11].

Medialization of the lamina papyracea (LP) is an interesting phenomenon that has been reported over the last 2 decades [11,12]. Posterior displacement of the globe has also been reported as a consequence of endoscopic sinus surgery (ESS) [11,12]. Cunnane et al. 2009 [11] documented five patients who developed medial bowing of the LP on follow-up CT scans after functional ESS, with a corresponding decrease in interorbital distance and 90% of those patients showing the globe positioned more posteriorly within the orbit. Reports suggest that in select cases, ESS-induced alterations of bony anatomy could extend beyond the sinonasal cavity to affect orbital structures. If pronounced, such changes could potentially cause orbital enlargement and vision disturbances.

Fortunately, clinically significant ocular complications from routine ESS are exceedingly rare; any LP medialization is typically small and asymptomatic, and overt enophthalmos or diplopia is seldom observed in the absence of an intraoperative orbital injury. In fact, other investigations have found little or no change in the LP after ESS for most patients [13]. While previous research has explored various outcomes of ESS, there are few data specifically addressing the long-term stability of structures like the inferior turbinate (IT) and lamina papyracea (LP) post-surgery. Our study aims to fill this gap by providing comprehensive insights into these anatomical changes over extended follow-up periods, thereby informing both surgical techniques and post-operative care strategies.

The true incidence and clinical significance of post-operative LP medialization remain uncertain, underscoring the need for further study. Given these considerations, the aim of this study is to investigate the effect of IT outfracture on the position of the IT and the duration of the effect, and to investigate if there are changes in the position of the LP and globe a while after complete ethmoidectomy.

## 2. Materials and Methods

This retrospective study was conducted in the Department of Otolaryngology—Head and Neck Surgery, Western University, London, Canada. The study protocol was approved by the Institutional Review Board ethics committee (WREM 112530, 1 April 2022). Electronic medical records of adult patients (>18 years) who underwent revision endoscopic sinus surgery (ESS) following a previous ESS for chronic rhinosinusitis between 2010–2020 at our institution were reviewed. Surgeries were performed by two fellowship-trained surgeons BR and LS. Patients were included in the study if they met the following criteria: 1. Patients underwent an initial primary ESS due to chronic rhinosinusitis, with a documented bilateral ethmoidectomy (anterior and posterior ethmoidectomy) and radiofrequency turbinoplasty with bilateral outfracturing of the inferior turbinate. 2. Patients underwent an initial computed tomography (CT) scan prior to the initial primary ESS with our department (Pre-ESS CT) 3. Patients underwent a second CT scan that was performed prior to a revision surgery (post-ESS CT). Patients were excluded if ESS was performed due to benign or malignant tumors, odontogenic or unilateral CRS.

### 2.1. Radiological Measurements

The inferior turbinate, globe and lamina papyracea positions were assessed using 0.5 mm axial and coronal CT scan slices, and were used to determine any changes between the baseline pre-ESS CT and post-ESS CT. An in-hospital PACS workstation was used to evaluate the CT scans (Vue Pacs, version 12.1.5.7014. Carestream Healthcare Inc., Rochester, NY, USA).

#### 2.1.1. Inferior Turbinate Position

**Figure 1:**
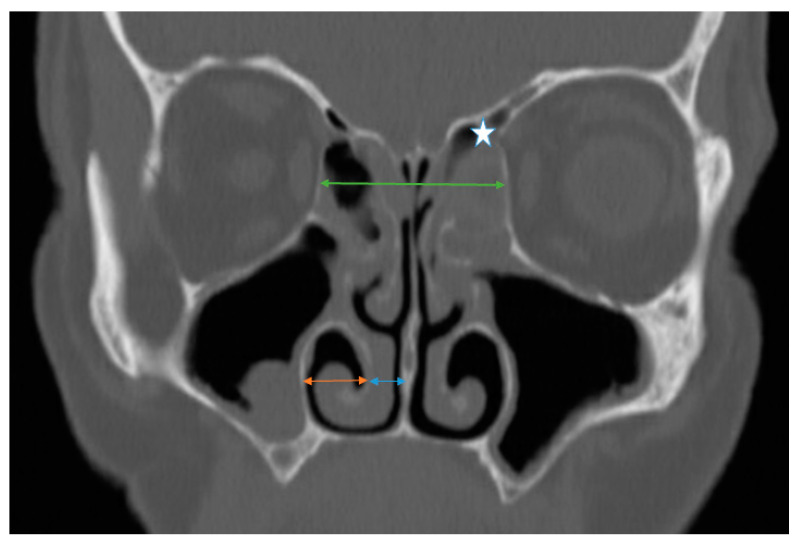
Sinus computed tomography scan, coronal plane. Star: the left anterior ethmoid artery. Green line: IOD in the coronal plane (IOD_Coronal_). Orange line: distance between the lateral border of the inferior turbinate to the lateral nasal wall (IT_L_). Blue line: distance between the medial border of the inferior turbinate and the septum.

To assess the radiological position of the inferior turbinate, several measurements were performed bilaterally using the CT scan (coronal plane). These measurements comprised the following: 1. The horizontal distance between the lateral border of the IT bone and the lateral nasal wall (IT_L_). 2. The distance between the medial border of the IT bone to the median nasal line (vertical line from the crista galli to the nasal spine) (IT_M_) (Figure 1). The point of entry of the anterior ethmoidal artery into the nasal cavity (nipple sign) served as an anchoring point to produce reproducible and accurate measurements in both the baseline and post-ESS CT images.

#### 2.1.2. Globe Position

**Figure 2:**
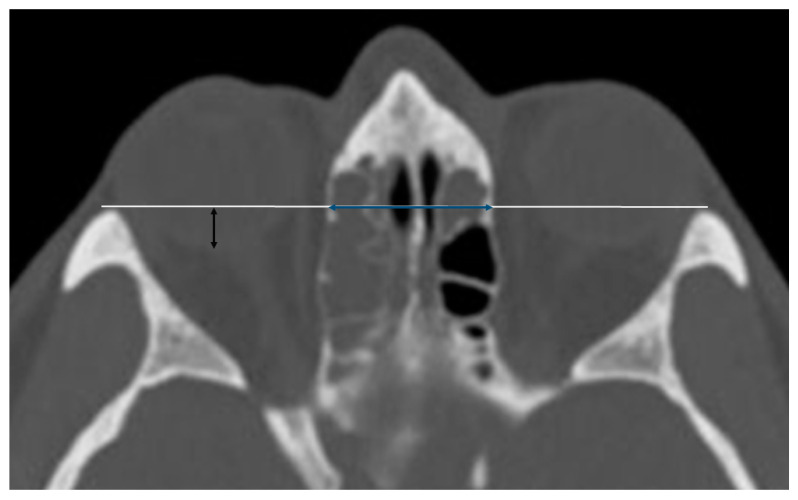
Sinus computed tomography scan, axial plane. White line: horizontal line drawn from the medial tip of the lateral orbital rim to the contralateral orbital rim. Blue line: distance between the two laminae papyracea in the axial plane (IOD_Axial_). Black line: represents the antero-posterior position of the right globe (PGP).

The antero-posterior position of the globe was measured in the axial plane on CT, using a horizontal line drawn from the medial tip of the lateral orbital rim to the contralateral orbital rim. The distance was measured from the horizontal line to the posterior edge of each globe passing through its midline (Figure 2).

#### 2.1.3. Lamina Papyracea (LP) Position

To investigate the effect of ethmoidectomy on the LP position we performed several measurements: in the coronal plane, a vertical line was drawn from the crista galli to the nasal floor, then a perpendicular line was drawn from one lamina papyracea reaching the contralateral lamina passing through the nasal septum at the level of the cribriform plate. The distance between the two laminae on the coronal CT scan was defined as the interorbital distance (IOD_coronal_). The point of entry of the anterior ethmoidal artery into the nasal cavity (nipple sign) served as an anchoring point to produce reproducible and accurate measurements in both the baseline and post-ESS CT images (Figure 1).

In the axial plane, using a horizontal line drawn from the medial tip of the lateral orbital rim to the contralateral orbital rim, we measured the distance between the two laminae, which was defined as interorbital distance (IOD_axial)_ (Figure 2).

#### 2.1.4. Time Effect

In order to investigate the effect of time on post ESS changes, we calculated the follow-up time (days)—elapsed between the ESS and post-op CT (follow-up CT). Then, we divided the participants’ follow-up time data into quartiles. A change in each variable (post–pre-ESS), measured as the delta, was calculated from each patient’s change in distance after surgical procedure (Δ).

### 2.2. Statistical Analysis

Descriptive and inferential analyses were performed using SPSS (v 20.0), with variables presented as mean and standard deviation. A paired *t*-test was used to compare the pre-operative and post-operative radiological measurements—results expressed as mean difference, *p*-value 95%CI and Cohen’s d effect size. The time effect analysis was performed using ANOVA comparison of variables’ Δ values.

## 3. Results

A total of 268 patients records were reviewed. From these, 44 met the inclusion criteria. There were 16 women and 28 men. Mean follow-up time was 1166 ± 603 days.

### 3.1. Inferior Turbinate Position

A total of 88 sides were measured (44 patients). Mean pre-ESS measurements of IT_L_ were 5.8 ± 1.6 mm, and IT_M_ were 7.6 ± 1.9 mm. Mean post-ESS measurements of IT_L_ were 4.5 ± 1.3 mm, and IT_M_ were 8.8 ± 1.8 mm. A statistically significant difference was found for the changes in both IT_L_ (95%CI 1.1 to 1.5; *p* < 0.0001) and IT_M_ (95%CI −1.5 to −1.1; *p* < 0.0001). (Table 1) The mean change of IT_L_ was −1.25 mm ±1.1, and 1.29 mm ± 1.04 for IT_M_.

### 3.2. Lamina Papyracea Position

Baseline and post-operative measurements were performed in all 44 patients, in both coronal and axial planes. Mean pre-ESS IOD_Axial_ was 25.1 ± 3.1 mm, and post-ESS IOD_Axial_ was 24.9 ± 3.4 mm. Mean pre-ESS IOD_Coronal_ was 25.4 ± 3.0 mm and post-ESS IOD_Coronal_ was 25.1 ± 3.2 mm. No significant statistical differences were seen in IOD_Axial_ and IOD_Coronal_ in pre-op and post-op CT scans. (*p* = 0.23 and *p* = 0.7, respectively).

### 3.3. Globe Position

A total of 88 pairs (pre- and post-op) were used for measurements. Mean pre-ESS measurements were 7.1 ± 2.0 mm, and post-ESS were 7.1 ± 1.9 mm. Surgery did not cause displacement of the globe in the antero-posterior direction (*p* = 0.915).

### 3.4. Time Effect Analysis

The first quartile comprised patients with a surgery follow-up time from 0 to <22 months, while the second group had a follow-up time of ≥22 to <35 months, the third ≥35 to <50 months and the fourth ≥50 months (n = 22 in each group). Both IT measurements (L, M) sustained their post-surgery changes throughout the four analyzed quartiles, with no significant changes between the four groups. Similarly, an absence of change in IOD and PGP variables post-surgery was noted in all four groups evenly.

## 4. Discussion

Inferior turbinate outfracturing can be performed during ESS to improve access to the middle meatus, or in addition to other reduction techniques to enlarge the nasal airway. It is considered to be a minimally invasive procedure, does not impair mucocilliary function and preserves the nasal epithelium.

Several studies have investigated the effect of IT outfracturing, and demonstrated a radiological lateralization on the inferior turbinate, and thus, improvement of the nasal airway [6,7,8,9]. However, all of these studies had a short follow-up ranging from 6–9 months. The durability of IT lateralization on IT position is debated. It was questioned first by Goode [14] in 1978. According to Goode’s clinical observations, the IT has a tendency to return to its original position.

**Figure 3:**
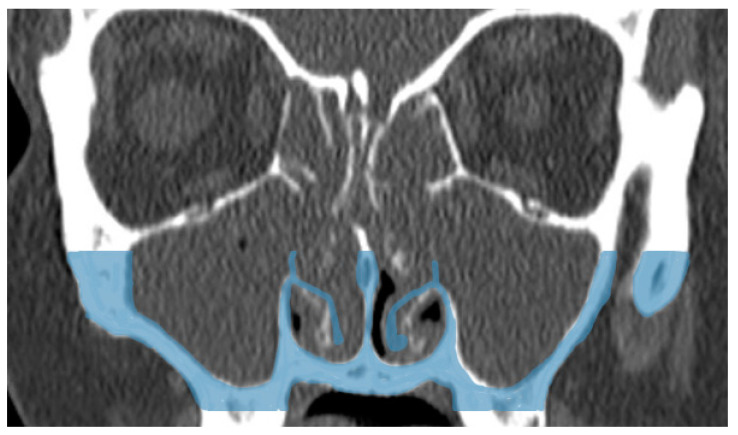
Showing merged computed tomography of pre-operative and post-operative images. Blue color shows the original position of the inferior turbinate prior to outfracturing.

The results of our study show that IT outfracturing is an effective method for the lateralization of the IT and enlarging the nasal airway. Importantly, it appears this effect is sustained for years (Figure 3).

Compensatory IT mucosal hypertrophy is generally found contralateral to a significantly deviated septum. Interestingly, mucosal hypertrophy of the medial aspect of the IT was documented after outfracturing [8]. This could significantly decrease the success rate of nasal surgery to improve nasal breathing. Thus, a combination of a soft tissue procedure (turbinoplasty) and a bony tissue procedure (outfracturing) may give better results in improving the nasal airway. This is supported by Karakurt et al. 2021 [15] who showed that radiofrequency ablation within an outfracture group had significantly better subjective results and better minimal cross-sectional area and nasal volume values than RF ablation performed only in a randomized trial. Additionally, Passàli et al. 1999 [16] showed that submucosal resection of the IT alongside outfracturing had better nasal outcomes than submucosal resection alone. Moreover, a recent systematic review showed that radio-frequency treatment of the inferior turbinates improved nasal obstruction based on patient-reported outcome measures with a sustained effect after several years [17].

The ethmoid air cell septa are thought to contribute to the resistance and endurance of the lamina papryacea, especially when exposed to abrupt hydraulic forces. This was supported by several authors [18,19,20].

Song et al. 2009 [19] found that patients with medial orbital wall fractures had a significantly lower number of ethmoid septa, and a larger lamina papyracea area supported per ethmoid air cell septum, than patients with inferior wall fractures. Similar findings were reported by Choi et al. 2015 [18], when 659 cases of blowout fractures were reviewed. It is stated that the number of ethmoidal air cell septa was lowest in the medial wall fracture group. Moreover, Sowerby et al. 2020 [20] performed an experimental cadaver study that showed that less energy is required to induce a fracture in the post-ESS side versus the control side. All of the above-mentioned studies shared the same pathophysiological mechanism, which is an abrupt traumatic mechanical force that led to a blowout fracture.

Other studies investigated the effect of ethmoidectomy on the position of the lamina papyracea and globes. In a small case-series by Cunnane et al. 2009 [11], they reported five patients with medial bowing of the lamina papyracea and 80% of them had posterior displacement of globes. Platt et al. [12] reviewed 100 patients post ESS, showing that 19% of them had a narrowing of their ethmoid cavity by 1–2 mm, 6% had a narrowing of more than 2 mm and 75% had no change (a change between (−1) to (+1)). A reported significant overall mean decrease in post-operative ethmoid width was only 0.6 mm. A major limitation was the accuracy and subjective selection of measurement points that can interfere with results, and cause a deviation of more than 2 mm. Visual symptoms are rare in ESS without orbital complication, thus, one would expect orbital complaints in cases of asymmetrical bowing of the lamina papyracea.

Our results showed there was no significant change in ethmoid width or the medialization of the lamina papyracea. Moreover, there was no significant alteration of globe position. Other studies have explored the effect of ethmoidectomy on the orbit with findings that align with ours. In a systematic review of the topic, no cases of intraoperative orbital were found, even though slight radiographic changes were occasionally noted [11]. Collectively, the evidence suggests that the orbit is far more resilient to sinus surgery than initially feared. Minor remodeling may occur, but it tends to remain clinically silent. Visual symptoms are exceedingly rare after ESS without an orbital complication, which matches our experience—none of our patients reported vision changes or noticeable cosmetic differences. Reasonably, if significant asymmetric LP bowing had occurred in any patient, they might experience orbital discomfort or diplopia, but no such cases were observed. Our results, therefore, support the conclusion that a standard ESS (including complete ethmoidectomy) is a safe procedure with respect to long-term orbital anatomy.

Our finding of essentially no change (Δ < 0.3 mm) in interorbital distance is not only statistically non-significant but also below the threshold of what most would consider a clinically relevant difference. It reinforces the notion that routine post-ESS orbital monitoring is unnecessary in the asymptomatic patient.

The main strength of our manuscript is the long follow-up compared to other published studies in the literature. Additionally, it included traditional patients who usually undergo a bilateral complete ethmoidectomy; patients with chronic rhinosinusitis.

This study’s limitations stem mainly from its retrospective design and its reliance on imaging conducted for clinical indications (revision surgery planning) rather than scheduled research follow-ups. There is a possibility of selection bias, as only patients who required revision ESS (and hence had a second CT scan) were included; however, this was mitigated by matching and the assumption that the need for revision was unrelated to turbinate/LP position. Measurement error is always a consideration in radiologic studies, though we attempted to minimize it as described. Another limitation is that post-operative CT scans were taken at the time of recurrence of the disease (prior to revision surgery), meaning the interval was dictated by when patients had persistent or recurrent CRS symptoms. It is conceivable that ongoing inflammation or polyp recurrence at the time of the second scan could have subtly influenced some measurements (for instance, a polyp in the ethmoid might exert pressure); however, given the lack of any systematic trend in our data, this effect seems negligible. Finally, we correlated anatomical changes with clinical inference but did not formally measure symptom scores in this retrospective review. Future prospective studies could pair objective measurements with symptom outcomes to further solidify the clinical significance of these anatomical findings.

## 5. Conclusions

In conclusion, inferior turbinate outfracture appears to have a durable effect on turbinate position that lasts for several years post-surgery. Patients can expect the enlarged nasal airway achieved intraoperatively to be largely preserved in the long term, which likely contributes to the sustained relief of nasal obstruction. Complete ethmoidectomy, in the absence of complications, did not cause medialization of the lamina papyracea nor did it alter the position of the globes in our series. The orbital contents and walls remain stable after ESS, supporting the safety of the procedure with respect to ocular anatomy. These results provide valuable evidence for surgeons counseling patients with chronic rhinosinusitis: the anatomical modifications achieved with ESS (including turbinate outfracture and sinus clearance) are not only effective but also long-lasting, without deleterious effects on adjacent orbital structures.

## Figures and Tables

**Table 1:** Inferior turbinate (IT) outfracture anatomical outcomes post endoscopic sinus surgery.

	Pre-ESS	Post-ESS				
	Mean (SD)	Mean (SD)	Mean Diff	*p*	95% CI	Cohen’s d
IT_L_ (*n* = 88; mm)	5.8 (1.6)	4.5 (1.3)	−1.2	<0.0001	−1.48 to −1.01	0.891
IT_M_ (*n* = 88; mm)	7.6 (1.9)	8.8 (1.8)	1.3	<0.0001	1.07 to 1.51	0.648
PGP (*n* = 88; mm)	7.1 (2)	7.1 (1.9)	0.3	−0.01	−0.2 to 0.18	0.001
IOD axial (*n* = 44; mm)	25.2 (3.1)	25.1 (3.1)	−0.1	0.17	−0.34 to 0.06	0.042
IOD coronal (*n* = 44; mm)	25.6 (3.1)	25.6 (3.0)	−0.0	0.85	−0.22 to 0.18	0.006

ESS: endoscopic sinus surgery; IT: inferior turbinate; IOD: intraorbital distance. PGP: posterior globe position. Paired *t*-test.

## Data Availability

The data underlying this article will be shared upon reasonable request to the corresponding author.
